# No attentional capture from invisible flicker

**DOI:** 10.1038/srep29296

**Published:** 2016-07-05

**Authors:** David Alais, Shannon M. Locke, Johahn Leung, Erik Van der Burg

**Affiliations:** 1 School of Psychology, University of Sydney, New South Wales, 2006 Australia; 2 Department of Psychology, New York University, New York, USA; 3 Department of Cognitive Psychology, Vrije Universiteit Amsterdam, Netherlands

## Abstract

We tested whether fast flicker can capture attention using eight flicker frequencies from 20–96 Hz, including several too high to be perceived (>50 Hz). Using a 480 Hz visual display rate, we presented smoothly sampled sinusoidal temporal modulations at: 20, 30, 40, 48, 60, 69, 80, and 96 Hz. We first established flicker detection rates for each frequency. Performance was at or near ceiling until 48 Hz and dropped sharply to chance level at 60 Hz and above. We then presented the same flickering stimuli as pre-cues in a visual search task containing five elements. Flicker location varied randomly and was therefore congruent with target location on 20% of trials. Comparing congruent and incongruent trials revealed a very strong congruency effect (faster search for cued targets) for all detectable frequencies (20–48 Hz) but no effect for faster flicker rates that were detected at chance. This pattern of results (obtained with brief flicker cues: 58 ms) was replicated for long flicker cues (1000 ms) intended to allow for entrainment to the flicker frequency. These results indicate that only visible flicker serves as an exogenous attentional cue and that flicker rates too high to be perceived are completely ineffective.

Human contrast sensitivity declines at high temporal frequency. Classical studies^1^,^2^ have shown that contrast sensitivity for rapidly modulating stimuli declines steeply for frequencies above 20 Hz. At 40 Hz, sensitivity is 100 times weaker than peak sensitivity and visibility of flickering stimuli ceases altogether at about 50 Hz^3^,^4^,^5^, a point known as the flicker fusion frequency. Above this rate, flicker is invisible and the percept becomes a time-averaged version of the stimulus. A contrast-reversing grating on a mean-luminance background, for example, would be completely indistinguishable from the background and would appear as a uniform gray surface. This occurs because neurons integrate input over time so that a stimulus cycling between positive and negative contrast will sum to zero when multiple cycles occur within the temporal integration period^6^,^7^.

The question arises of whether flicker too high to be perceived can nonetheless have perceptual effects. It is a tantalising question because while cells in primary visual cortex exhibit responses to modulating stimuli up to ~50 Hz^8^, corresponding well with the perceptual flicker limit, the response range in the lateral geniculate nucleus extends considerably higher^9^. A number of recent papers have looked at whether imperceptibly high flicker can produce perceptual effects^10^,^11^,^12^,^13^,^14^. Shady reported that perceptual sensitivity to flicker at the upper end of the visible range was raised following adaptation to flicker too fast to be perceived. This was explained in terms of a series of temporal filters, with the filter mediating flicker perception being preceded by an adaptable filter with a higher temporal limit, a framework that maps well onto the higher response range of pre-cortical neurons relative to cortical neurons.

Another study claimed that a cue flickering invisibly at 50 Hz could guide attentional selection to that location and improve discrimination of a target subsequently presented at the same location^10^. The authors accounted for this in terms of the invisible flickering stimulus entraining neural oscillations at the same frequency in visual cortex. Their rationale was that this would benefit attentional selection because gamma-band (~30–70 Hz) oscillations in neural activity are observed when observers do top-down attentional tasks. Whether this study’s results can be explained in terms of stimulus-entrained gamma oscillations is open to debate. First, it is not at all clear that a stimulus modulating at imperceptibly high rates would entrain gamma-band oscillations in the relevant cortical neurons. Second, even if their stimulus did entrain such oscillations, it is questionable whether they would be functionally equivalent to those generated endogenously in top-down, voluntary attention tasks. Finally, the role of gamma-band neural oscillations in attention and other perceptual tasks has been hotly debated and it is not clear whether they play a causal role or are a non-causal epiphenomenon^15^,^16^,^17^,^18^.

A more fundamental question concerns the nature of the stimulus used in this study^10^. Using a cathode ray tube (CRT) video monitor to present their 50 Hz flicker is problematic. Typically, CRT monitors will refresh their displays at rates no higher than 100 or 120 Hz, meaning a visual temporal frequency above the flicker threshold can only be created by alternating between two stimuli on every frame. This A-B alternation can produce a nominally 50 or 60 Hz modulation at refresh rates of 100 and 120 Hz, respectively, but it is far from a clean signal. Such a stimulus would have abrupt transitions in intensity, creating frequency splatter at higher frequencies. In addition, any spatial or temporal non-linearity in the display device, including non-linearities in the display’s luminance output, could create visible low-frequency components. Another potential problem is that the temporal ramping in this experiment at stimulus onset and offset could potentially create DC shifts that would alter mean luminance, effectively creating a contrast between the stimulus and background that may be visible.

Because the claim that imperceptibly fast flicker can nonetheless modulate attentional processing has strong theoretical implications, and given the critical importance of stimulus control in validly drawing such a conclusion, we sought to repeat a similar version of this experiment using a visual display with a very high refresh rate of 480 Hz and a carefully linearised luminance output. At this stimulus refresh frequency, an invisible visual flicker of 60 Hz, well above the flicker fusion frequency and in the middle of the gamma-frequency band, could be presented as a clean, sinusoidal modulation sampled evenly and symmetrically at eight points per cycle rather than a crude triangular sampling at two points per cycle. To preview the experiments, we measured flicker detection at eight temporal frequencies (20, 30, 40, 48, 60, 69, 80 and 96 Hz) and verified that detection performance was at chance level at 60 Hz and higher and therefore that our stimuli were free of visible artefacts. We then tested whether the same flicker rates would capture visual attention when presented as a cue just prior to a visual search task. Whether cues were presented briefly (58 ms) or for a longer entrainment period (1000 ms), our results show no evidence at all that invisible flicker can cue attention to a target location. In contrast, when the flicker was visible, strong attentional cueing effects were observed.

## Experiment 1

**Figure 1:**
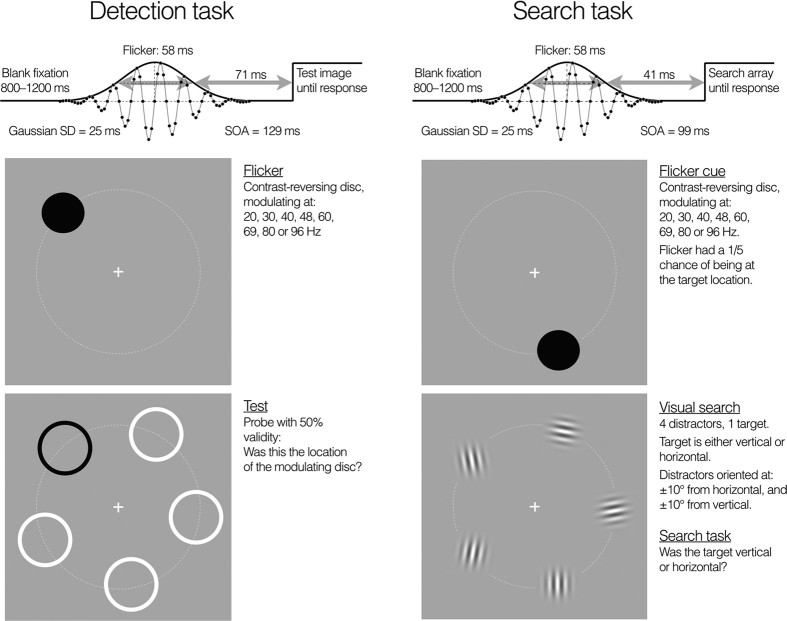
Flicker detection (left-hand side) was measured for a circular disc at one of five locations at 8 temporal frequencies: 20, 30, 40, 48, 60, 69, 80 and 96 Hz. Trials began with a variable blank fixation period (800–1200 ms) followed by a Gaussian-windowed temporal modulation. The Gaussian standard deviation was 25 ms, giving a flicker duration of 58 ms when defined by the full width at half height. The modulation shown is a 60 Hz sinusoid, with each cycle defined by 8 time points at the 480 Hz frame rate. A 71 ms blank followed the flicker (timed from the half-height) then a test pattern containing five rings appeared and subjects indicated whether the black ring corresponded to the flicker location (2AFC). The black ring indicated the flicker location in half the trials or one of the other four locations in the other half, randomly determined. The display was then adapted to test whether the flickering disc captured attention prior to a visual search task (right-hand side). Spatial and temporal parameters were nearly identical to the detection task except that the test pattern was a five-element visual search display and the blank period was 41 ms, making an SOA of 99 ms. The target was either vertical or horizontal, and the four distractors were ±10° from vertical and horizontal, and subjects searched for the target and indicated its orientation. The flicker cue location varied so that it cued target location on 20% of trials and was incongruent on 80% of trials. Search times for congruent and incongruent trials were contrasted to test for a congruency effect. Any temporal frequency that captures attention should produce a significant congruency effect.

Experiment 1 involved a flicker detection task, as shown in the left-hand side of Fig. 1. Flicker detection was measured at eight temporal frequencies ranging from 20 Hz to 96 Hz and the same flicker stimulus was then used as a cue for a visual search task (right-hand side of Fig. 1).

### Methods

#### Ethic Statement

The experiment was approved by the Human Research Ethics Committee of the University of Sydney. The experimental procedure conformed to the declaration of Helsinki and participants gave informed consent prior to commencing the experiment.

#### Participants

18 participants took part in the experiment (12 female, age range 18–26 years). All participants had normal or corrected-to-normal vision, and were naïve as to the purpose of the experiment.

#### Apparatus

The experiment was conducted in a dark room where participants were seated at a desk facing a matte white PVC projector screen (Epson ELP-SC21B, 1771 × 996 mm) from a distance of 171 cm. A PROPixx DLP colour projector was located in front of the participant just below the line of sight and cast a viewable image area of 129 × 72 cm (43 × 24 degrees of visual angle) at a native resolution of 1920 × 1080 pixels at 120 Hz. The projector was connected via a DataPixx2 display driver and controlled using software written in MATLAB 2014a (The MathWorks, Inc.) running on an Apple Mac Pro computer. The projector had a linearised luminance output and when running in quadrant mode displayed images with a frame rate of 480 Hz at a resolution of 960 × 540 pixels.

#### Stimuli

All stimuli, whether for the flicker detection task or the visual search task, were equally spaced at five points on a virtual circle with a radius of 7.3 degrees centred on a white fixation cross (see Fig. 1). From trial to trial, the five stimulus locations were randomly rotated around the circle so that their positions varied unpredictably but remained equally spaced.

Flicker detection trials and visual search trials both began with a contrast-reversing flicker stimulus (Fig. 1, top panels) which cycled over time according to a sinusoidal pattern. The flicker stimulus was a hard-edged circular disc of uniform luminance and 2.8° width which modulated through positive and negative luminance relative to the average-luminance background (76.3 cd m^−2^). Maximum and minimum luminances were 152.9 and 0.21 cd m^−2^ and contrast was set to 80% of this range. Ten different modulation temporal frequencies were compared: 20, 30, 40, 48, 60, 69, 80 and 96 Hz. The contrast modulation was generated by sampling at 480 Hz from a sine wave at a given flicker frequency and flickering stimulus was temporally windowed by a Gaussian function with a standard deviation of 25 ms, giving a flicker cue duration of 58 ms where duration is defined as the full-width of the Gaussian at half height.

In flicker detection trials, the flicker stimulus was followed by a blank interval of 71 ms (timed from the point where flicker offset reached half-height) and then a test array of five rings (Fig. 1, lower left panel). The flicker/test SOA was therefore 129 ms. One ring always surrounded the flicker cue’s location with the other four equally spaced around the virtual circle. Four of the rings were black and one white. There was a 50% chance of the white ring surrounding the flicker cue’s location and a 50% chance of the white ring surrounding one of the other four locations. The rings were larger than the flickering disc (3.3° diameter) so as not to spatially overlap the flicker cue, and the blank interval prior to the test array ensured there was no temporal overlap. The task was to respond whether or not the white ring indicated the location of the flicker cue and the rings remained visible until a response was made.

Visual search trials began the same way as the flicker detection trials, with a disc stimulus flickering for 58 ms (Fig. 1, upper right panel) followed by a blank period of 41 ms. After the blank period a visual search display (Fig. 1, lower right panel) was presented (cue/search display SOA = 99 ms) which contained five Gabor patches spaced equally around a virtual circle, one being the target and four being distractors. The target Gabor was either vertical (0°) or horizontal (90°), whereas the four distractor Gabors were randomly allocated (without replacement) orientations that were offset from vertical or horizontal by 10 degrees: −10°, +10°, 80° or 100°. All Gabors had a contrast of 50%, a spatial frequency of 5.4 cyc deg^−1^ and were windowed by a two-dimensional Gaussian with standard deviation of 0.43 degrees, giving the Gabors a full-width at half-maximum of 1.01°. The Gabors were above zero contrast out to a diameter of 3.1°. The flickering disc preceding the search display was an uninformative cue to target location as it was equally likely to occur at any of the five Gabor locations, and so only indicated the target with 20% validity. The task was to make a speeded response indicating whether the target was vertical or horizontal (which alternated randomly over trials) and the search display remained visible until a response was made.

**Figure 2:**
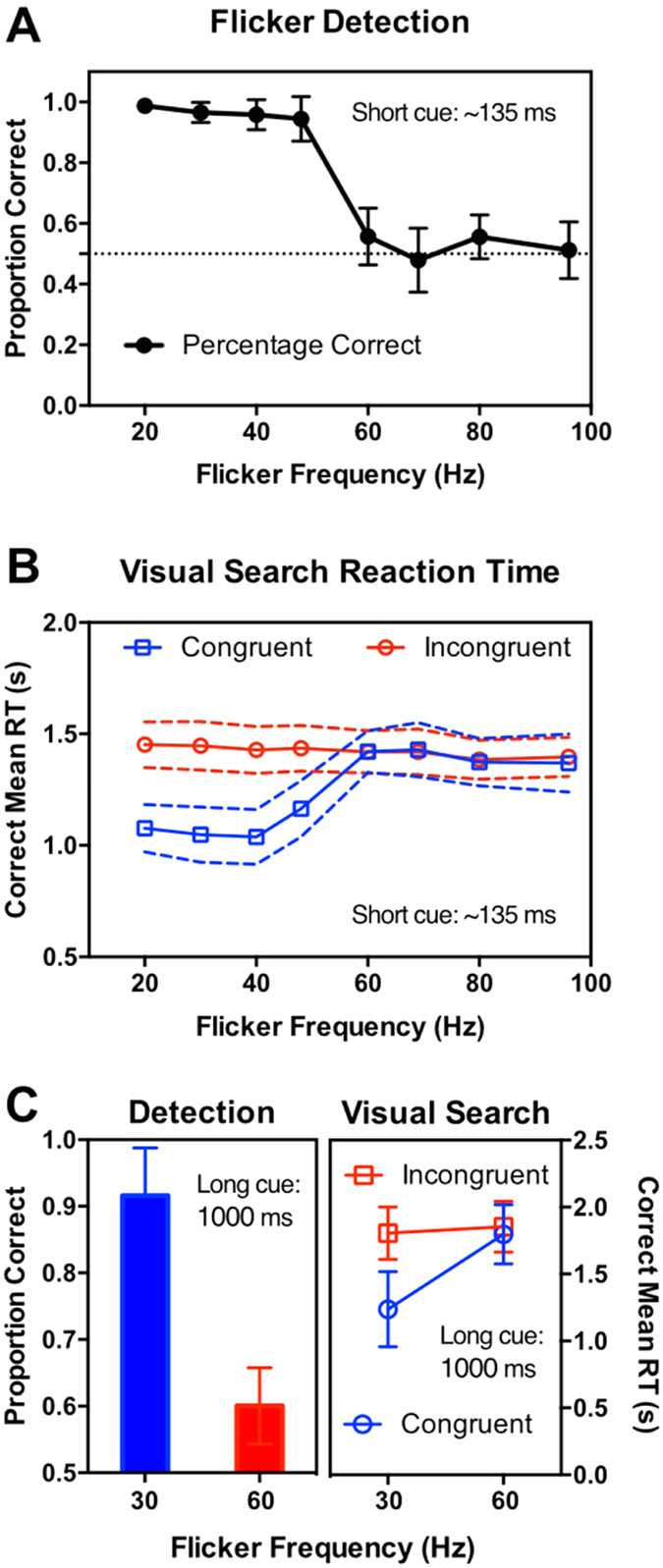
Results from Experiment 1 showing group mean data for 18 participants. All error bars show 95% confidence intervals. (**A**) Flicker detection performance as a function of temporal frequency for a disc modulating sinusoidally over time. Performance was very close to ceiling for 20, 30, 40 and 48 Hz and dropped sharply to chance level for 60, 69, 80 and 96 Hz. (**B**) Visual search data showing mean correct search times for targets at the flicker-cued location (congruent) or non-cued locations (incongruent). For the four visible flicker frequencies (20, 30, 40 and 48 Hz), there is a large congruency effect with valid cues speeding search times by an average of 358 ms relative to incongruent cues. For the four invisible flicker frequencies (60, 69, 80 and 96 Hz), search times did not differ significantly between congruent and incongruent cues (average difference = 12 ms). (**C**) Results for flicker detection (left) and flicker-cued visual search using a 1000 ms flicker period, the duration employed by Bauer *et al*.^10^. Results replicate the data for short-duration cues shown in part **A** and **B** of this figure. For detection, performance was near ceiling for 30 Hz and near chance-level for 60 Hz; for visual search, there was a strong congruency effect for 30 Hz flicker but not for 60 Hz flicker.

The data shown in Fig. 2C came from replications of the detection and visual search experiments that were identical in all respects except that the cue duration was extended to 1000 ms (to match the flicker duration in Bauer *et al.’s* study^10^), and only two frequencies were tested: 30 and 60 Hz.

### Design and procedure

Participants began by doing the flicker detection conditions to establish their ability to detect the temporally modulating disc. In a mixed design, the eight temporal frequencies were presented 32 times each in a randomised order making a total of 256 trials. As shown in Fig. 1 (lower left panel), each flicker presentation was followed by a test array of five rings, four white and one black. On 50% of trials the black ring surrounded the location of the flickering disc and on the remaining trials the black ring was distributed among the other four locations. In an unspeeded task where accuracy was stressed, participants made a 2AFC response, indicating whether or not the black ring indicated the location of the flickering disc or not, guessing if necessary and error feedback was provided to help optimise their performance. Despite the 5 possible locations, presenting the black ring at a given location made the task a binary choice and thus the 50% valid cue was neutral and offered no strategic advantage, as was explained to participants prior to the experiment. The dependent variable was proportion correct performance. Each trial was separated by an inter-trial interval varying randomly between 800 and 1200 ms and self-paced pauses were provided every 16 trials.

Once flicker detection performance was established, participants did the visual search experiment. Visual search trials began with a flickering disc (58 ms) at one of the eight temporal frequencies used in the flicker detection task. Following a blank screen presented for 41 ms, a visual search array of five Gabor stimuli was presented (Fig. 1, lower right panel). The search target was a Gabor that was either vertical or horizontal, while the other four Gabors were distractors having orientations ±10° from vertical and ±10° from horizontal. In a speeded task, participants searched for the target and responded whether it was vertical or horizontal. The target’s orientation was randomly vertical or horizontal over trials with equal probability, while all four distractor orientations were present on each trial. The spatial locations of the 5 stimuli were randomly redistributed on every trial. Trials were separated by an interval varying randomly between 800 and 1200 ms and self-paced pauses were provided every 50 trials. 100 trials were collected for each temporal frequency, making a total of 800 trials, completed in two sessions of 400. The dependent variable was mean search time for correctly searched trials and error feedback was given. The location of the flicker stimulus preceding the search array varied randomly over trials among the five locations of the search array. All locations had a 20% probability of being cued by the flicker and the cue was therefore uninformative about the target’s location, as was explained to participants prior to the experiment. We used this to calculate a congruency effect for each flicker frequency by comparing search times for the 20% of trials that were validly cued by the flicker with search times for the 80% of trials that were invalidly cued. To the extent that the flicker cue is effective, there should be a speeding up of search times in validly cued trials compared to the invalidly cued trials.

### Results

#### Detection performance

Group mean data for detecting the flickering disc as a function of temporal frequency are shown in Fig. 2A. A one-way, repeated-measures analysis of variance (ANOVA) confirmed that performance depended strongly on temporal frequency, *F*(7, 119) = 188, *p* < 0.0001. As expected detection performance for the lower temporal frequencies was high as these frequencies are well below the flicker fusion limit (~50 Hz) while performance for the higher temporal frequencies was close to chance level. The transition in performance from ceiling to chance is strikingly abrupt, with performance at 48 Hz still very high at 0.944 while performance at 60 Hz is 0.557 and not significantly above chance. Either side of this transition, performance was very consistent, being very near ceiling for 20, 30, 40 and 48 Hz (mean = 0.964), and being very close to chance level for 60, 69, 80 and 96 Hz (mean = 0.526). Confirming this, post hoc t-tests with Bonferroni correction showed detection performance for flicker frequencies of 20, 30, 40 and 48 Hz were all significantly above chance (all ps < 0.0001) whereas performance for all higher flicker rates (60, 69, 80 and 96 Hz) was not significantly different from chance.

Overall, the data clearly demonstrate that flicker detection performance for our stimulus conditions segregates into two very clear categories: ‘visible’ flicker for modulation rates of 48 Hz and below, and ‘invisible’ flicker for modulation rates of 60 Hz and above. This strong segregation sets up clear-cut predictions for the congruency effects expected in our visual search condition. If only visible flicker can exogenously cue attention, congruency effects should be limited to 20, 30, 40 and 48 Hz and be absent for all higher frequencies.

#### Search performance

Group mean data for the visual search task as a function of temporal frequency are shown in Fig. 2B. This experiment was virtually identical to the flicker detection task in all respects except the test pattern was replaced by a visual search array (see Fig. 1, right-hand side). The figure plots group mean correct reaction times as a function of the temporal frequency of the flickering disc, which cued the target location on 20% of trials and one of the distractor locations on 80% of trials. Data for these two kinds of trial are plotted separately as ‘congruent’ (when the flickering disc cued the target) or ‘incongruent’ (the flicker cued a distractor). An ANOVA on mean correct RT was conducted with cue congruency and flicker frequency as within subject variables. The ANOVA yielded highly significant main effects for both factors: cue congruency *F*(1, 272) = 50, *p *< 0.0001, and cue frequency *F*(7, 272) = 4.7, *p *< 0.0001. The congruency effect was expected because the flicker stimulus just prior to the search display was designed to provide a cue that would capture exogenous attention and either shorten search times if the flicker cued the target’s location or lengthen search times if the flicker drew attention to a distractor. Averaged over all temporal frequencies, search times were 1240 ms for congruent trials and 1422 ms for incongruent, a congruency effect of 182 ms.

Our particular interest is whether the congruency effect occurs over all temporal frequencies or whether the search benefit is limited to the visible frequencies determined by the flicker detection data. The ANOVA revealed a very strong interaction between cue congruency and flicker frequency *F*(7, 272) = 7.0, *p* < 0.0001, confirming that the congruency effect does depend on temporal frequency. Comparing the upper and lower panels in Fig. 2 shows strikingly that the search benefit from congruent flicker cues is limited to the visible flicker frequencies shown in Fig. 2A. This was statistically confirmed with Bonferroni-corrected two-tailed t-tests showing very strong congruency effects for the four lower temporal frequencies: 20, 30 and 40 Hz, *ps* < 0.0001; 48 Hz, *p* = 0.0019). On average over these frequencies participants responded faster by 358 ms (standard error = 29 ms) when the cue was congruent with the target compared to when it was not. There were no significant congruency effects for any of the invisible frequencies (60, 69, 80 and 96 Hz, all *ps* > 0.05), indicating that invisible flicker does not guide attention.

One key difference between our study and Bauer *et al.’s* study^10^ is that our cues were brief (58 ms). Bauer *et al*. used 1000 ms flicker durations, reasoning that this would provide time to entrain neural modulations at the flicker frequency and thus mimic the internally-generated gamma-band activity observed during attentional selection. We therefore repeated our visual search experiment using a flicker duration of 1000 ms to see if cues presented for longer periods could facilitate visual search. We first compared detection of 30 and 60 Hz flicker in an experiment identical to the one just described except for the longer flicker duration. We then repeated the visual search experiment, exactly as described above except that only 30 and 60 Hz flicker were compared and the flicker cue was presented for 1000 ms.

Results are shown in Fig. 2C for both detection (left) and search (right) and are virtually identical to those reported for the brief flicker duration in Fig. 2A,B. Detection was near ceiling for 30 Hz flicker, and near chance-level for 60 Hz flicker, with the difference significant on a two-tailed paired t-test, t9 = 10.09, p < 0.0001. The visual search data were analysed as above with a two-way (frequency × congruency) repeated-measures ANOVA which revealed significant main effects of flicker frequency *F*(1, 18) = 6.4, *p* = 0.0206, and cue congruency *F*(1, 18) = 18.0, *p* < 0.0005. Critically, as observed for the short-duration cue, there was a strong interaction between frequency and cue congruence *F*(1, 18) = 12.0, *p* = 0.0028. Post-hoc comparisons with Sidak’s correction show that the congruency effect is significant at 30 Hz (t18 = 5.4, p < 0.0001) but not at 60 Hz (t18 = 0.54, p = 0.8351). These results confirm the first finding that invisible flicker plays no role in cueing attention, despite the long entrainment period, and rules out differing flicker durations as an explanation.

**Figure 3:**
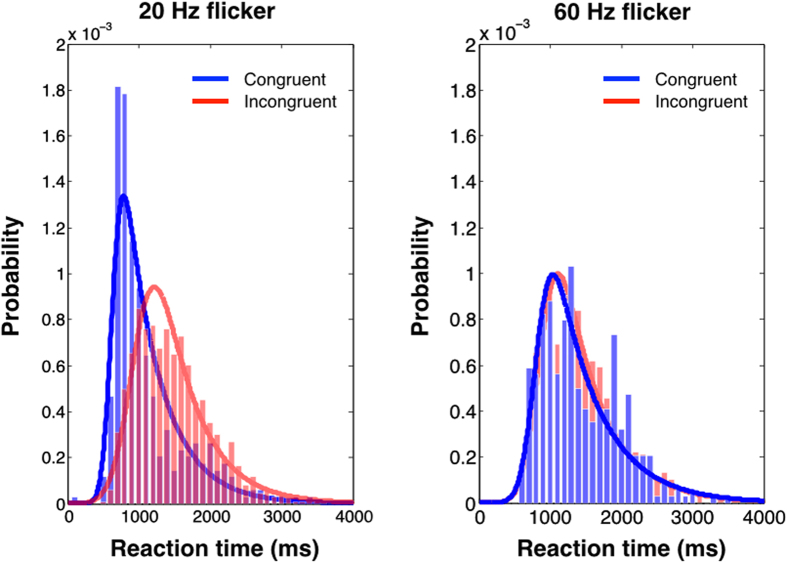
Histograms showing distributions of reaction times for the visual search task. Data from all observers were pooled and binned into 100 ms bins. Blue data show search times for trials preceded by a congruent flicker cue (i.e., cuing target location), red data show search times from incongruent flicker cues. Continuous lines show best fitting log-normal distributions.

To look closer at the congruency effect we pooled the data over all observers and compiled distributions of search times for congruent and incongruent trials for every flicker frequency. Figure 3 shows the distributions for congruent and incongruent cues overlaid for 20 Hz (left-hand side) and for 60 Hz (right-hand side). In the 60 Hz condition it is clear that both distributions are virtually identical, regardless of cue congruency, not only in the location of the peak but in bandwidth. This was typical of all the invisible flicker frequencies. Comparing the fit parameters across the 60, 69, 80 and 96 Hz flicker conditions with a two-tailed paired t-test showed that the congruent and incongruent distributions did not differ significantly in their mean (*t(3)* = 1.472, *p* = 0.234) or bandwidth (*t(3)* = 0.958, *p* = 0.409). In contrast, the 20 Hz condition shows clearly differing distributions for congruent and incongruent cues, with the congruent distribution’s peak shifted towards shorter reaction times and having a narrower bandwidth. The same t-tests were run on the fit parameters across the 20, 30, 40 and 48 Hz flicker conditions and showed that the congruent distributions had a much lower mean than the incongruent distributions (*t(3)* = 57.280, *p* < 0.0001) and a narrower bandwidth (*t(3)* = 18.170, *p* = 0.0004). Overall, for visible flicker frequencies, search times rise very steeply in the congruent condition, showing the efficacy of the cue with search times clustered near a floor value of efficient search. The incongruent distribution has a much shallower rise and a broader upper tail, consistent with a serial search.

**Figure 4:**
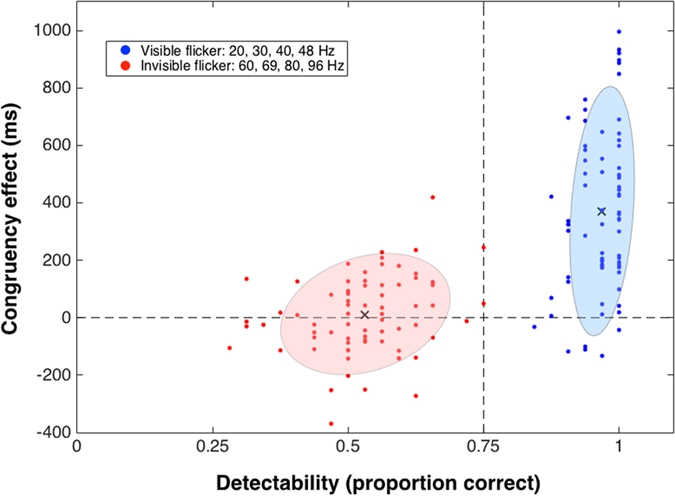
Data showing the relationship between the magnitude of the congruency effect and flicker cue detectability, for every subject at each of the eight flicker rates. The ellipses show results from a cluster analysis using a Gaussian mixture model, with the cross showing the centroid of the Gaussian and the shading showing the extent of the central 95% of the distribution. The vertical dashed line shows threshold level for the flicker detection data. As summarised by the centroids of the clusters, mean detectability for the invisible frequencies was at chance level and produced no congruency effect, while for the visible frequencies detection was at ceiling and mean congruency effect approached 400 ms.

Figure 4 plots, for each temporal frequency and observer, the relationship between the visibility of the flicker cue measured in the flicker detection task and the corresponding congruency effect measured in the visual search task. Each data point represents an individual participant’s detection level for a given flicker frequency on the x-axis and the magnitude of the corresponding congruency effect on the y-axis. The 144 data points (18 participants × 8 temporal frequencies) cluster into two very distinct clouds, reflecting the clear segregation of both the detectability data (see Fig. 2A) and the congruency effect (Fig. 2B) into two levels based on temporal frequency. The four lowest flicker rates (visible frequencies) had high levels of detectability (group mean = 0.97) and produced large congruency effects (group mean = 369 ms), as summarised by the central cross in the right-hand cluster. The four highest flicker rates (invisible frequencies) had low levels of detectability (group mean = 0.53) and produced no significant congruency effect (group mean = 9 ms), as summarised by the central cross in the left-hand cluster.

**Figure 5:**
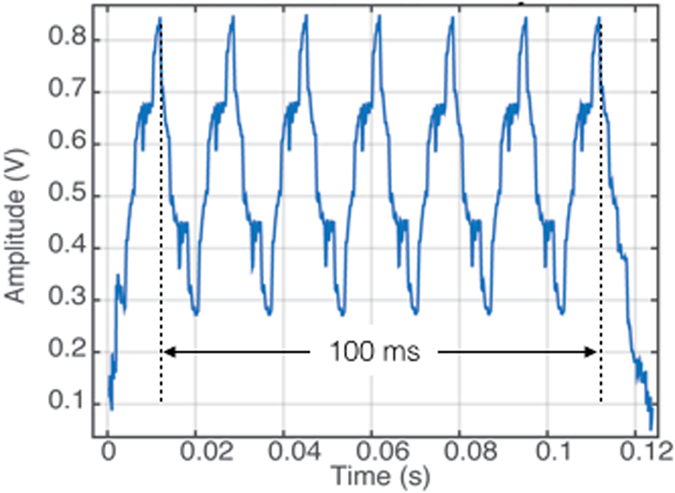
Flicker stimulus as recorded directly from the 480 Hz projector used to present the stimuli in these experiments. The plot shows seven overlaid traces recorded on a high speed digital oscilloscope sampling at 100 MHz, sampled by a photodiode with a response time of 13 nanoseconds. The overlaid samples show the signal is highly reproducible and that the timing is stable and accurate, with six full samples spanning 100 ms at 60 Hz.

Our findings seem clearly to show there is no attentional capture effect for invisibly high flicker rates. To be sure of this conclusion, however, we directly measured the signals presented to our observers to verify that the stimulus was correctly modulating, even when flickering imperceptably fast. For this purpose we used a photodiode with an ultrafast response time of 13 nanoseconds (Advanced Photonics PDB-C107) and recorded directly from the visual projector using a high-speed digital oscilloscope (Siglent SHS810, 100 MHz, 1Giga samples/s). Figure 5 shows a trace of six cycles of the 60 Hz flicker stimulus recorded directly from the projector using the photodiode attached to the projection screen. The signal is clearly cyclical and completes six cycles in 100 ms. Verifying the output signal in this way is critical for precisely characterising the stimulus. Interestingly, none of the papers investigating the attentional effects of invisibly high flicker have done a frequency analysis of their signal output^10^,^11^,^14^.

## Experiment 2

Experiment 1 showed that flicker detection was at or near ceiling for all frequencies up to and including 48 Hz and then dropped sharply to chance level at 60 Hz and above. Experiment 2 replicates the 48 and 60 Hz conditions from Experiment 1 and includes an intermediate frequency of 53 Hz to better characterise the transition between visible and invisible frequencies. The design of Experiment 2 combines both tasks from Experiment 1 into a combined ‘search and detect’ task so that we can measure both detectability and the congruency effect at the three frequencies, with particular interest in the 53 Hz condition.

### Methods

Methods were very similar to Experiment 1 in all respects except that only 3 flicker frequencies were used (48, 53, 60 Hz) and a combined ‘search and detect’ task was used (with spatial and temporal parameters as for Experiment 1, see Fig. 1). Following a flicker cue at one of the three temporal frequencies, participants searched for the target (a vertical or horizontal grating) in an array of 5 gratings in a speeded search task and indicated the target’s orientation. Immediately after their response for the search task, five rings appeared (four black, one white) and participants indicated whether the white ring corresponded to the flicker location (which it did on 20% of trials) or not, guessing if necessary. Based on the power of the first experiment, a sample of 10 people from the same university student population served as participants.

### Results

#### Detection performance

**Figure 6:**
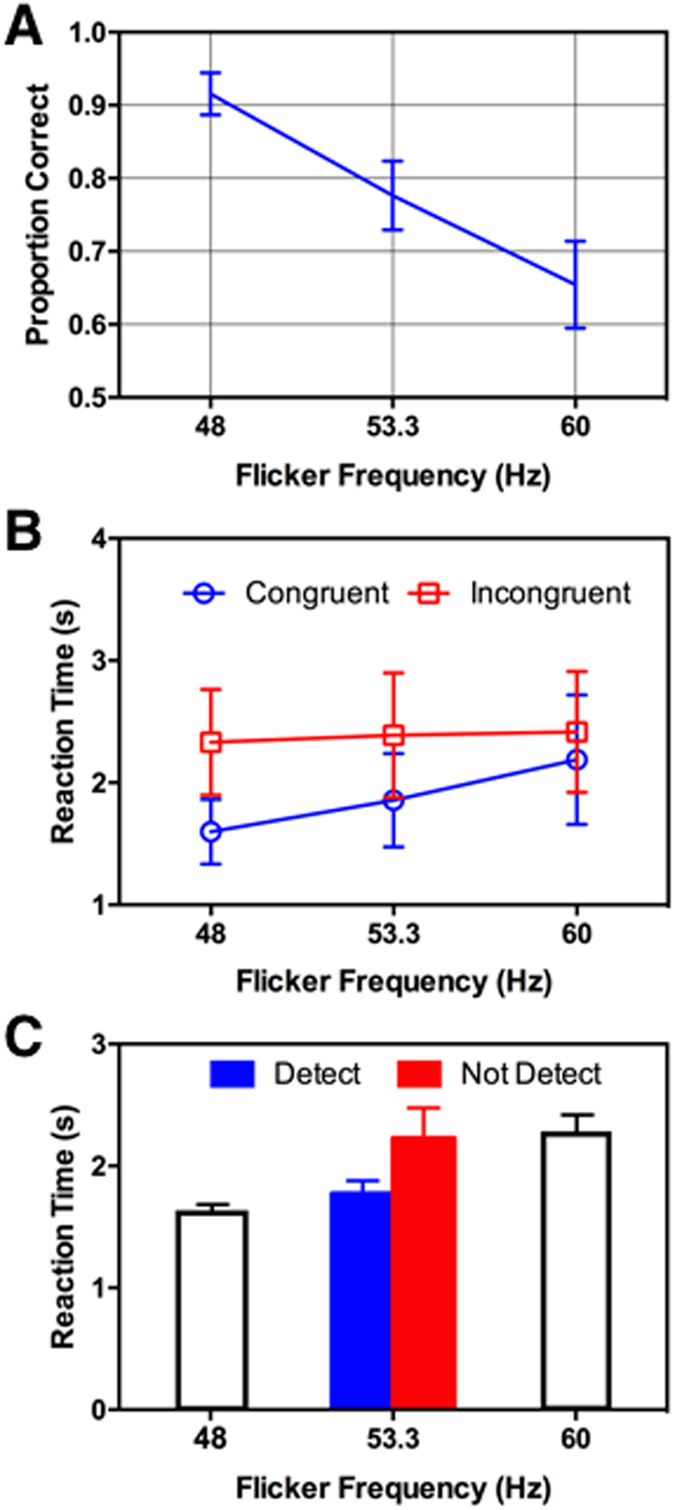
Results from Experiment 2 showing data for 10 participants. All error bars are 95% confidence intervals. (**A**) Flicker detection performance as a function of temporal frequency. Performance for 48 and 60 Hz agree closely with the same frequencies in Experiment 1, with 53 Hz close to threshold level (0.75). (**B**) Visual search data showing mean correct search times as a function of temporal frequency, for targets at flicker-cued (congruent) or non-cued (incongruent) locations. The interaction between frequency and congruency was significant, confirming the dependency of the congruency effect on frequency seen in Experiment 1. (**C**) Trials from the 53 Hz condition were divided into two groups based on whether the flicker was visible or not. When detected, the flicker cue sped search times to a level similar to that seen with 48 Hz flicker, and when not detected, search times slowed to that seen with 60 Hz flicker. These data confirm that flicker must be perceived in order to speed visual search.

Group mean flicker detection performance is shown in Fig. 6A and visual search performance in 6B. The detection data was analysed in a one-way, repeated-measures ANOVA and confirmed that flicker detection performance depended strongly on temporal frequency, *F*(2, 18) = 36.42, *p* < 0.0001. Detection performance for the two replicated frequencies closely matched the values obtained in Experiment 1 (48 Hz: 0.94 vs 0.91; 60 Hz: 0.56 vs 0.60, estimates from Experiments 1 and 2, respectively) with detection for 53 Hz flicker intermediate between the two other frequencies and very close to threshold at 0.77.

#### Search performance

Group mean data for the visual search task are shown in Fig. 6B. Data for the 20% of trials in which the flicker cue indicated the target location are plotted as ‘congruent’ and the remaining 80% of trials as ‘incongruent’ (the flicker cued a distractor). An ANOVA on mean correct RT was conducted with cue congruency and flicker frequency as within subject variables. The ANOVA yielded no significant main effect of temporal frequency *F*(2, 27) = 0.83, *p* = 0.447, but a significant effect of cue congruency *F*(1, 27) = 42, *p* < 0.0001. Most relevant for this experiment, there was a significant interaction between cue congruency and flicker frequency *F*(2, 27) = 3.6, *p* = 0.04, confirming again that the congruency effect depends on temporal frequency, as in Experiment 1, and that it occurs over the narrower range tested in Experiment 2.

Because Experiment 2 involved a dual-task, search-and-detect design, we were able to do a *post hoc* analysis on the congruency effect as function of whether the flicker cue was successfully detected or not. In practice, the 48 Hz flicker was almost always correctly detected (91% of trials) and the 60 Hz flicker was detected at near-chance level (60% of trials), however detection of the 53 Hz flicker cue was at threshold level (77%). Because flicker in the 53 Hz condition was about equally likely to be detected or not, we could test whether search RTs in that condition depended on whether the cue was visible or not. To do so, we calculated the mean correct search RT in all 53 Hz trials in which the flicker location was correctly identified (and presumably visible) and compared it to mean RT for trials in which the flicker location was incorrect (presumably invisible). As shown in Fig. 6C, mean RTs in these two categories were significantly different, *t*(383) = 3.70, *p* < 0.0001, with RTs being longer and similar to the 60 Hz condition when flicker was not visible, and shorter and similar to the 48 Hz condition when flicker was visible. Indeed there was no statistical difference between RTs for 60 Hz and the ‘invisible’ 53 Hz flicker, *t*(460) = 0.056, *p* = 0.956. Although the 53 Hz RT for visible trials was significantly less than for invisible, it was slightly higher than for 48 Hz and just reached significance, *t*(689) = 2.41, *p* = 0.016 (α = 0.017, Bonferroni corrected).

### General Discussion

We investigated flicker detection rates for discs modulating at various temporal frequencies ranging from 20 to 96 Hz that were smoothly rendered at a presentation rate of 480 Hz. We then used these stimuli as cues to determine which flicker rates were able to capture attention in a visual search paradigm. Flicker detection was at or near ceiling for frequencies of 20, 30, 40 and 48 Hz and dropped sharply to chance level at 60, 69, 80 and 96 Hz. When these flicker rates were used as cues just prior to visual search trials, discs modulating at detectable rates (20–48 Hz) were highly effective cues, as evidenced by the strong congruency effect: search times were much faster when the cue indicated the target location than when it cued a distractor location. In contrast, there was no congruency effect for the four invisible flicker frequencies (60–96 Hz): search times were equivalent regardless of whether the flicker cued the target or a distractor. Our findings provide no support at all for the recent claim that flicker at invisibly high rates can guide attentional selection^10^,^11^,^12^. Our data suggest that flicker that is too high to be perceived is completely ineffective as an attentional cue. Experiment 2, with the inclusion of a flicker frequency of 53 Hz that was detectable at threshold level, supports this conclusion by showing that flicker did capture attention in trials where the flicker was detected, but not for trials where the flicker was undetected.

In addition to the much smoother temporal sampling afforded by our 480 Hz display, we believe this discrepancy might also be explained by three significant methodological improvements we implemented in seeking to replicate Bauer *et al.’s*^10^ original study. First, we did not cue spatial location by flickering the target stimulus itself (a Gabor patch, in their experiment). Instead we cued attention by modulating a disc stimulus at the target location and only presented the target grating after a 41 ms blank period. The advantage of this is that the target grating was never modulated or altered in any way that could draw attention to it, meaning any effect must be due to the pre-cue and not to a change in the target itself. Second, our search task did not involve responding to a stimulus change in the target. In Bauer *et al.’s* experiment, the target Gabor underwent a spatial frequency change, whereas we used a compound task in which subjects first had to locate the target location and then make a forced-choice judgment about the target’s orientation^19^,^20^. This has the benefit that there is no manipulation of the target which could in itself attract attention. The spatial frequency change they used was 0.14 cpd (7% of the baseline spatial frequency), which was both detectable in itself (i.e., to make the task possible) and was presented for long enough – 600 ms – for eye movements or a reallocation of attention to the target location and thereby produce an apparent cue congruency effect. There was a similar problem in another of their experiments in which the target reversed contrast each 100 ms during the 600 ms test period, and involved the addition of a dot probe – which would add luminance to the target and again help make it visible. For these reasons, we did not conduct an exact replication of Bauer *et al.’s* study^10^. In our design we preferred never to change the target during the test period when subjects were looking for the target so any difference among the conditions can only be attributed to the allocation of attention and its congruency with the target location. We still test the central claim of Bauer *et al.’s* study^10^ and we do so in a tight design which shows very clear congruency effects at all visible frequencies and none otherwise.

A third methodological problem in Bauer *et al.’s* study^10^ is that they used only three elements in their test display (one target, two distractors), but cued the target with 50% validity. This is a problem because if the target has a 33% probability of detection, cueing the target with a 50% validity biases the cue to the target location and makes the cue informative^21^, meaning there is a performance benefit to using the cue strategically to guide responses. In our study, each stimulus in the five-element test array was equally likely to be cued (20%), meaning the cue did not have a bias to the target location^22^ and subjects were instructed that there was no performance gain to be had by using the cue. Finally, we adopted Van Diepen *et al.’s*^14^ modification of Bauer *et al.’s* procedure by including a blank interval between the flicker pre-cue and presentation of the search array. Van Diepen *et al*. observed that an immediate transition from flicker to target produced a visible flash which served to cue the target. By introducing a blank they eliminated this transition flash and observed a halving of the cueing effect. We adopted this measure, as well as using temporally smoothed stimuli, and eliminated all trace of a visible flash.

Even if these concerns about stimulus presentation and methodology are set aside, Bauer *et al.’s* interpretation of their results seems questionable. Their main conclusion is that a cue flickering at an invisible gamma rate works by entraining internal gamma modulations, even though the cue does not engage awareness. However, the durations of their flicker cues were rather long: the congruency effect they report (i.e., faster responses to the cued target) is not evident at 100 ms or 200 ms cue duration, but only occurs after 300 or 400 ms of cueing. The problem with this time course is that 300 ms is long enough for eye movements and endogenous attention to search for the flicker cue, which it could do if it contained visible components. Bauer *et al.’s* interpretation is that it is an exogenous effect but requires and entrainment period of at least 300 ms to build up the neural oscillations. If there were any visible component, 300–400 ms is long enough to move the eyes and foveating the flicker cue would enhance any visible component and make the endogenous interpretation more likely. By contrast, our cue duration (58 ms) and cue/test SOA (99 ms) were much shorter to prevent attentional shifts or eye movements. In our design, any congruency effect can be attributed to the flicker being an effective exogenous cue, and this was only observed for visible flicker.

Another important point when examining a potential role for invisible flicker is to consider the fidelity of the signal generated by the display device. Whereas our 60 Hz stimulus clearly showed sinusoid-like signal traces (Fig. 5), the same cannot be said of the 50 Hz signals used in previous studies that were generated on CRT monitors running at a frame rate of 100 Hz. Signal fidelity is important if conclusions of critical theoretical significance are to be drawn based on the assertion that particular temporal frequencies are ‘invisible’ yet still have perceptual or attentional consequences. None of the previous studies claiming a role of invisible flicker has measured the *actual* stimulus output, although Van Diepen^14^ did present an FFT of an *ideal* simulation of Bauer *et al.’s* stimulus (a vector in which the alternating cue luminance was specified for each millisecond over a 2 s period). A number of neurophysiological studies have shown that refresh rate is a factor that affects neural response latencies in visual cortex and that detectable responses linked to typical CRT refresh rates can be observed in neural recordings^23^,^24^. Our stimulus avoids these limitations because the sampling rate was so high at 480 Hz and much closer to real-world visual stimulation, which is continuous rather than being composed of a rapid series of luminance transients as is the case for CRT stimuli.

Although our evidence suggests that invisibly high flicker rates cannot cue attention, several studies have shown that adaptation to invisible stimuli can nonetheless elevate detection thresholds of visible stimuli. Flicker that is too fast to be perceived can raise thresholds at lower rates than are perceptible^13^,^25^, and in a similar vein, spatial frequencies too high to be perceived can induce threshold changes at lower, visible spatial frequencies^26^. These and other studies show evidence of basic detection thresholds being influenced by invisible targets and imply implicit processing of the invisible adapting stimuli^27^. One interpretation of these results is that the adapting neural activation is too weak to activate conscious perception but strong enough to induce adaptation, presumably in neurons tuned to the upper end of the spatial and temporal frequency range whose tuning bandwidth extends to those higher, non-perceptible frequencies, and that appropriately designed experiments can demonstrate these threshold elevations at lower frequencies in the perceptible range. However, demonstrating adaptation effects at visible frequencies is conceptually very different from proposing that the invisible frequencies can directly influence attention at those frequencies. For attention to be allocated volitionally (consciously) to a stimulus, it would by definition need to be visible, and as for exogenous attention, our own data here clearly show that a stimulus cannot capture unless it is perceptible. This dissociation between sensory perception and conscious awareness is supported by many binocular rivalry studies showing that rivalry suppression of an adapting stimulus does not always reduce its efficacy in adapting aftereffects, suggesting that conscious awareness is occurring at a processing stage subsequent to that mediating adaptation^28^,^29^.

In sum, we find no support for the claim that invisible flicker is able to engage attentional selection^10^. We conclude that only visible flicker can serve as an attentional cue. We base this on two lines of evidence, first, the observation that cues flickering at rates too high to be perceived cannot be detected and do not produce a congruency effect in a search task, and second, on the basis of our rigorously controlled and verified visual signals. We suggest a simpler explanation of Bauer *et al.’s* finding is that it was due to a combination of methodological and stimulus limitations that allowed unintended artefacts to determine their results.

## Additional Information

**How to cite this article**: Alais, D. *et al*. No attentional capture from invisible flicker. *Sci. Rep.* **6**, 29296; doi: 10.1038/srep29296 (2016).
